# Long-Term Care Insurance Policy Intensity and the Policy Matching Degree of Pilot Cities in China

**DOI:** 10.1093/geroni/igab046.1934

**Published:** 2021-12-17

**Authors:** Rong Peng, Bei Wu

**Affiliations:** 1 School of Economics, Guangzhou, Guangdong, China (People's Republic); 2 New York University, New York, New York, United States

## Abstract

Since China launched long-term care insurance (LTCI) pilot in 15 cities in 2016, the LTCI policy schemes have been developed gradually. Based on the 61 LTCI policy documents, this study evaluated the LTCI policies intensity in each pilot area by building a PMC index model with 10 primary variables and 44 two-level variables. Using the coupled coordination model, the coordination development indexes were calculated to evaluate the level of matching between LTCI policies and economy development and population structure. The results showed that the PMC index valued between 0.646 and 0.922. The three cities having the highest level of policy strength (PMC>0.8) were as follows: Qingdao, Nantong, and Jingmen. The three cities having the lowest level (PMC<0.7) were Ningbo, Qiqihar and Chongqing. The indexes of the coordination degree varied from 0.263 to 0.594. Shanghai, Qingdao and Guangzhou had the highest level of coordination degree, while Anqing, Chende and Qiqihaer are the lowest .The difference of financing mechanism of pilot cities was one of the main determinants of policy intensity. The matching degree was relatively low. Qingdao was a unique city having both high policy intensity and matching degree. It was suggested that the intensity and matching degree of LTCI policies should be improved to develop a national LTCI system in China.

